# The SAPPHIRE criteria, history of myocardial infarction and diabetes predict adverse outcomes following carotid endarterectomy similar to stenting

**DOI:** 10.1007/s00392-019-01546-3

**Published:** 2019-09-25

**Authors:** Roland Richard Macharzina, Carolin Müller, Matthias Vogt, Steven R. Messé, Werner Vach, Thomas Winker, Michael Weinbeck, Matthias Siepe, Martin Czerny, Franz-Josef Neumann, Thomas Zeller

**Affiliations:** 1 Department of Cardiology and Angiology II, University Heart Center Freiburg-Bad Krozingen, Suedring 15, 79189 Bad Krozingen, Germany; 2 Department of Surgery, Ortenau Klinikum Lahr, Lahr, Germany; 3 Department of Cardiovascular Surgery, University Heart Center Freiburg-Bad Krozingen, Bad Krozingen, Germany; 4 Department of Neurology, University of Pennsylvania, Philadelphia, USA; 5 Functional Biomechanics Laboratory, University Hospital Basel, Spitalstrasse 21, 4031 Basel, Switzerland; 6 Institute of Neurology, University Heart Center Freiburg-Bad Krozingen, Bad Krozingen, Germany

**Keywords:** Carotid artery stenosis, Endarterectomy, Stroke, Myocardial infarction, Diabetes, SAPPHIRE trial

## Abstract

**Aims:**

Identifying factors associated with worse outcome following carotid endarterectomy (CEA) is important to improve prevention of major adverse cardiovascular and cerebrovascular events (MACCE), yet rarely used for registries. We intended to identify predictors of MACCE following CEA as recently analysed for stenting.

**Methods and results:**

Patients undergoing CEA at 2 centers over 13 years were entered into a database. Baseline clinical characteristics, procedural factors and a panel of clinical and lesion-related high-risk features (SHR) and exclusion criteria (SE), empirically compiled for stratification in the SAPPHIRE trial, were differentially analysed using Cox regressions. The analysis included 748 operations; 262 (35%) asymptomatic, 208 (28%) with previous strokes, and 278 (37%) with transient ischemic attacks (TIA). The overall 30-day MACCE rate was 6.7%, 5.0% in asymptomatic and 7.6% in symptomatic patients. Previous MI (HR 2.045, *p* = 0.022), diabetes (HR 2.111, *p* = 0.011) and symptomatic patients (HR 2.045, *p* = 0.044) were independently associated with MACCE. SE patients (*n* = 81) had a MACCE rate of 13.6%; the MACCE rate of the remainder dropped to 5.8% (4.7% in asymptomatic and 6.5% in symptomatic patients). Hazard ratio for SHR patients was 2.069 (CI 1.087–3.941) and 2.389 for SE (CI 1.223–4.666), each compared to all patients with lower risk and adjusted for symptomatic status. Among SHR and SE criteria NYHA 3–4, contralateral occlusions and intraluminal thrombus were significant determinants and MI < 4 weeks before CEA showed a strong trend (*p* = 0.05).

**Conclusion:**

Patients identified by SHR and SE criteria, prior MI and diabetes warrant increased attention to prevent MACCE following CEA.

**Graphic abstract:**

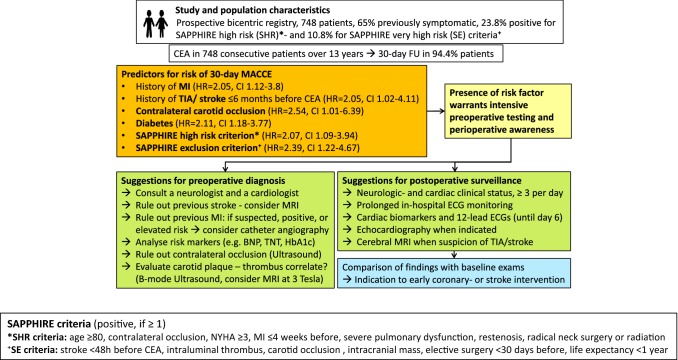

**Electronic supplementary material:**

The online version of this article (10.1007/s00392-019-01546-3) contains supplementary material, which is available to authorized users.

## Introduction

Carotid endarterectomy (CEA) is an established treatment strategy for many patients with carotid artery stenosis. Several randomized controlled studies and registry studies proved its effectiveness in carefully selected patient populations still arising controversies about improved medical treatment alone in subgroups of patients, requiring further comparative randomized trials to be answered [1–4]. The decision-making on treatment options can be challenging, as the clinician needs to translate the results of randomized controlled studies with highly selected patient groups to “real-life” clinical practice and to take the individual risk of additional non-neurological reasons of worse outcomes like myocardial infarctions (MI) into account, to discuss the option of best medical treatment versus the necessity, feasibility and longer-term benefit of the revascularization procedure bearing cardioembolic sources of recurrent stroke in mind [5–8]. Correspondingly, several potential risks of worse outcomes such as history of MI were mostly not explored in previous randomized trials at nowadays standards having partially excluded patients at an increased risk, such as having suffered a recent MI, an evolving stroke or a stroke of larger size [1–6]. Intending to further reduce the individual periprocedural risk, a single-arm real-world registry study can serve to discover and validate preprocedural factors and to warrant more intense postoperative monitoring of patients at a specific risk. Risk predictors may need to be further analysed in strata of future comparative randomized trials on treatment modalities.

Some registry studies have focused on identifying factors associated with a higher perioperative risk [9–16]. However, in these studies, the primary combined outcome of interest was death and stroke. Recent RCTs and large registries for quality assessment and improvement focused on the combined major adverse cardiovascular and cerebrovascular (MACCE) endpoint which incorporates myocardial infarction (MI), death and stroke as recently reviewed [5, 6]. Some of these studies have emphasized the importance of perioperative MI as an endpoint since it has been demonstrated to be a predictor of mortality [17–20]. The aim of this study is to identify independent risk factors for MACCE with special emphasis on clinical and specific lesion related predictors as empirically compiled by the Stenting and Angioplasty with Protection in Patients at High Risk for Endarterectomy (SAPPHIRE) trial investigators in patients who have undergone CEA in a real-world clinical practice setting (detailed criteria see Table 4) [17]*.*

Previously, we established the importance of the SAPPHIRE high risk and exclusion criteria on outcomes following carotid stenting [21]. However, SAPPHIRE inclusion and particularly the exclusion criteria have not been systematically evaluated for the contemporary definition of MACCE at 30 days following CEA [6, 21–27]. We intended to prospectively assess whether the high risk and exclusion criteria from the SAPPHIRE study (detailed in Table 4) are associated with 30-day MACCE outcomes in addition to other potential predictors.

## Methods

### Enrollment and data collection

Prospectively acquired data were analysed from consecutive patients who underwent CEA at two centers; the University of Freiburg and the Heart Center Bad Krozingen over a 13 years period.

**Fig. 1:**
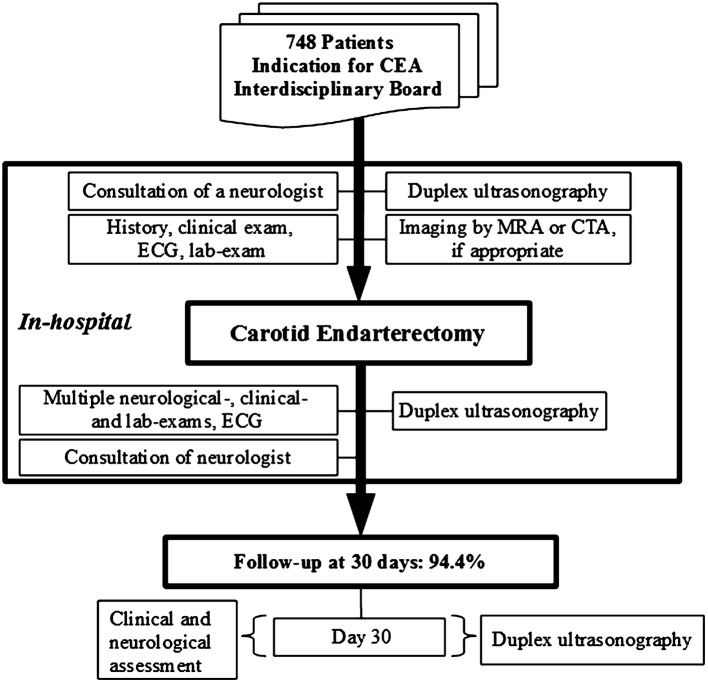
Study design. *CEA* carotid endarterectomy, *CTA* computed tomography angiography, *ECG* electrocardiogram, *MRA* magnetic resonance angiography

Inclusion of patients and the decision to undergo endarterectomy was made upon consensus of a neurologist, a vascular surgeon and a radiologist, adhering to contemporary recommendations [6, 22, 26]. Major inclusion criteria of patients were internal carotid artery stenosis (ICAS) of ≥ 50% in symptomatic and ≥ 70% in asymptomatic patients [6]. Patients planned for combined open heart surgery and CEA were excluded. A neurologist examined all patients before, after the operation and before discharge. Data of the clinical assessment including vascular risk factors, symptomatic status and duplex ultrasonographic (DUS) stenosis grading were systematically documented using case registration forms for this pre-planned analysis (Fig. 1). ICAS was defined symptomatic if it had caused a TIA, amaurosis fugax or stroke in the past 6 months. Stroke was defined as focal neurologic deficit due to a vascular occlusion in the territory of the ICAS, lasting more than 24 h, or with confirmation of acute infarct on neuroimaging. If the neurological deficit lasted < 24 h, and no infarct was seen on neuroimaging, a TIA was diagnosed. Stroke severity was evaluated according to the modified Rankin Scale (mRS). MI was defined by enzyme abnormalities plus symptoms or ST-segment changes according to contemporary standards [6, 18, 27]. For this prespecified analysis, the patients were assigned into risk-groups according to the criteria defined by the SAPPHIRE trial [17, 23]. The SLR (SAPPHIRE low risk) group were typical patients with no specific risk factors, the SHR (SAPPHIRE high risk) group were patients at high risk including age ≥ 80, severe cardiac or pulmonary disease, restenosis and contralateral internal carotid artery occlusion and the SE (SAPPHIRE exclusion criteria) group comprised patients who would have been excluded from SAPPHIRE, such as those presenting an intraluminal thrombus, having suffered a stroke within the preceding 48 h or having a life expectancy < 1 year (Table 4) [17]. A detailed list is available in the supplemental material. The study was approved by the ethics committee of the Albert-Ludwigs-University Freiburg.

### Patient evaluation and data processing

DUS was performed by experienced sonographers and the NASCET-defined degree of ICAS was determined using internally validated classification criteria [28].

### Peri- and post-procedural medication

Heparin was administered intraoperatively at therapeutic dosage, adjusted to body weight (100 IU/kg). Aspirin was continuously administered 100 mg daily, beginning prior to the surgery. Antihypertensive drugs and statins were prescribed according to updated treatment recommendations and adherence to drugs was controlled at follow-up visits.

### Operation and postsurgical examinations

CEA was performed under general anaesthesia. The technical strategy by classical endarterectomy or eversion operation was adjusted to the individual anatomic findings. Resection of any part of the stenotic segment was performed without or with an interponate, when reconstruction was not possible or in cases of vessel kinking, independent of the classical thrombendarterectomy versus eversion strategy applied. Technical failure was detected if the plaque could not be removed as in the case of far distal location. Operation time was categorized at 75th percentile [29]. Patients were monitored for neurological and cardiovascular symptoms every 3 h on an intermediate care unit for at least 24 h after the operation and a neurological examination was carried out at day 3 and again prior to discharge.

### Follow-up and endpoint assessment

Patients were asked to return to an out-patient follow-up visit consisting of a neurological and cardiovascular exam and a DUS of carotid arteries scheduled at day 30 post-procedure. Postoperative endpoint data of patients unable to attend the follow-up visit were abstracted from hospital charts and documents requested from general practitioners, neurologists and cardiologists. If follow-up was missing, the patient was censored at his last visit. The major combined endpoint MACCE consisted of stroke, MI or death within 30 days of the procedure. Every event was included. In case of multiple events per patient, only the first event was counted.

### Statistics

SPSS (version 23.0; Chicago, IL, USA) was used for analysis. Surgical, patient- and procedure-related variables were analysed by Cox regression (Tables 2, 3, 5 and Supplementary Tables 1, 2). Significant risk factors identified on univariate analysis (*p* < 0.05) and outcome predictors as found in other studies such as age, contralateral occlusion and diabetes mellitus were entered into multivariate regression (Table 3). The predictive power of a score containing factors found on multivariate analysis was assessed by receiver operating characteristic analysis. Continuous data were compared by Student’s *T* test. Significance was assumed at *p* < 0.05.

## Results

### Patient population and baseline characteristics

Seven hundred and forty-eight consecutive patients with carotid artery stenosis underwent CEA, 184 (24.6%) being women, having a mean age of 69 ± 9 years. Additional baseline characteristics are displayed in Table 1. In total 486 (65%) patients had suffered an ipsilateral cerebrovascular event in the past 6 months before the operation, of which 208 were strokes, 278 were TIAs (77 patients with transient monocular blindness included). Among patients with prior stroke, the mRS was rated 3 or higher in 19.7% before the operation. The mean time from neurological events to the operation was 30 (± 36) days (27 ± 32 days for stroke versus 32 ± 40 days for TIA, *p* = 0.455). Three (0.4%) patients were operated on recurrent stenosis after previous CEA and five patients on recurrent stenosis after CAS (0.7%). Following the risk categories of the SAPPHIRE study, 178 (23.8%) patients met the high-risk criteria and 81 (10.8%) patients would have been excluded from the SAPPHIRE study due to expected very high risk.

**Table 1:** Baseline and procedure related characteristics

	*n* (%)
Patients	748
Age (years)	
Mean (± SD)	69.3 (± 9.1)
Range	41–90
≥ 80	97 (13.0)
Sex (male/female)	564 (75.4) / 184 (24.6)
Asymptomatic (ipsilateral)	262 (35.0)
Grade of stenosis (%-NASCET)	
High grade (≥70)	619 (82.8)
Moderate (50–69)	118 (15.8)
Low grade (<50)	11 (1.5)
Contralateral occlusion	51 (6.8)
Body-mass-index [kg/m^2^] (± SD, range)	26.9 (± 4.14; 16.0–49.0)
Diabetes mellitus	218 (29.1)
Hypertension	657 (87.8)
HLP on statin treatment	559 (74.8)
Smoking	400 (53.5)
Coronary heart disease	297 (39.7)
Myocardial infarction	130 (17.4)
Heart failure NYHA (III, IV)	64 (8.6)
Renal failure (NKF III, IV):	214 (28.6)
Atrial fibrillation	55 (7.5)
First-degree atrio ventricular-block	78 (10.4)
Family history of cardiovascular disease	183 (24.5)
Antiplatelet therapy	681 (91.2)
Eversion endarterectomy	453 (61.0)
Resection^a^	68 (9.1)
Operation time (± SD, range)	84.1 (±34.34 20.0–305.0)

*HLP* hyperlipoproteinemia, *NKF* National Kidney Foundation, *NYHA* New York Heart Association heart failure scale, *n* number of patients, *SD* standard deviation

^a^Resection is defined as any resection independent of the operation strategy used

### Acute CEA results

Overall technical success was achieved in 733 patients (97.9%). In 10 patients (1.3%) the stenosis could not be operated on due to anatomic reasons not revealed by preoperative DUS and missing a preoperative angiography. In six patients (0.8%) detailed data were missing.

### Observed events

Follow-up at 30 days was completed for 94.4% of the population. Overall, 50 patients (6.7%) experienced a perioperative MACCE event within 30 days of the procedure, of which 41 (5.5%) were strokes, 8 (1.1%) were MIs and one patient died (0.1%). Table 2 shows the event rates depending on symptomatic status. Most of these events (*n* = 39, 78%) happened within post-operative day 3 and 20 (40%) were occurring on the day of the operation. Sixty-one percent of all strokes were disabling (mRS ≥ 3; *n* = 25; mean 3.22 ± 1.42, range 1–6). MI’s were mostly NSTEMIs (87.5%; mean time to event 2.9 ± 3.2 days, range 1–10). Left ventricular function deteriorated in two cases after perioperative MI. Myocardial biomarkers of patients with MI were Troponin T [mean ± SD (range) 2.03 ± 1.94 (0.07–5.7) (ng/ml), CK 819.6 ± 432.8 (130–1296) (U/l) and CK-MB 106.9 ± 53.6 (33–172) (U/l)].

**Table 2:** Observed events in patients with and without history of neurologic events up to 30 days after CEA

Event	30 days (*n* = 748)		Symptomatic (*n* = 486)		Asymptomatic (*n* = 262)		HR	CI	*p*
	*n*	%	*n*	%	*n*	%			
Death	1	0.1	1	0.2	0	0	n.a	n.a	n.a
Stroke	41	5.5	33	6.8	8	3.1	2.241	1.035–4.852	0.041
Ipsilateral Stroke	40	5.3	33	6.8	7	2.7	2.561	1.133–5.790	0.024
Myocardialinfarction	8	1.1	3	0.6	5	1.9	0.327	0.078–1.370	0.126
Death and stroke	42	5.6	34	7.0	8	3.1	2.309	1.069–4.988	0.033
MACCE	50	6.7	37	7.6	13	5.0	1.547	0.823–2.911	0.176

*CI* confidence interval, *HR* hazard ratio, *p* level of significance for difference of event rates between the symptomatic and asymptomatic group, *MACCE* Major Cardiac and Cerebrovascular Events, *n* number of patients, *n.a.* not applicable

### Identification of patient- and procedure-related risk factors

Baseline characteristics, risk factors and procedure-related factors were analysed for their influence on MACCE in a univariate Cox analysis (Table 3 shows selected data, for detailed list see Supplementary Table 1). Diabetes, coronary heart disease (CHD), previous myocardial infarction, first-degree AV block and any resection, mostly resulting from elongated vessels (*n* = 58, 85% of all resections) were significant predictors for the MACCE endpoint. A Cox regression subanalysis for the endpoint MI showed a significant association with CHD (HR 10.7, CI 1.3–8.7, *p* = 0.027), SHR and SE criteria (HR 9.8, CI 2–48.5, *p* = 0.005), hyperlipidemia (HR 7.3, CI 1.8–30.7, *p* = 0.006), NYHA ≥ 2 (HR 6.6, CI 1.3–32.5, *p* = 0.021), diabetes (HR 4.2, CI 1–17.6, *p* = 0.049) and age (HR 1.1, CI 1–1.2, *p* = 0.029).

**Table 3:** Uni- and multivariate Cox analysis of predictors for MACCE at 30 days (*n* = 748)

	Univariate			Multivariate		
	HR	95% CI	*p*	HR	95% CI	*p*
Age	1.019	0.988–1.052	0.235	1.012	0.979–1.047	0.466
Contralateral occlusion	1.927	0.821–4.522	0.132	2.542	1.010–6.396	0.047
Symptomatic	1.547	0.823–2.911	0.176	2.045	1.018–4.109	0.044
Myocardial infarction	2.295	1.267–4.157	0.006	2.045	1.108–3.777	0.022
Diabetes	2.127	1.219–3.709	0.008	2.111	1.183–3.767	0.011
Resection	2.300	1.116–4.074	0.024	2.264	1.082–4.736	0.030
First-degree atrio ventricular-block	2.230	1.115–4.460	0.023	1.965	0.964–4.007	0.063

*CI* confidence interval, *HR* hazard ratio, *MACCE* Major Cardiac and Cerebrovascular Events, *p* level of significance

When the various rationales for resection were assessed separately, severely visually altered, heterogenous, irregular and calcified vessel wall pathomorphologies that made reconstructions complicated had a higher MACCE rate (HR 7.377, CI 2.651–20.532, *p* = 0.0001), while resections for mere elongation, kinking or coiling had not (HR 1.359, CI 0.539–3.426, *p* = 0.516). There was no difference in outcome if the surgeon was experienced (i.e. more than 50 prior CEAs) or not (HR 0.803; CI 0.443–0.455; *p* = 0.47).

### Identification of independent risk factors

Factors with significant hazard ratios on univariate analysis and factors established in prior studies including age, symptomatic status, and contralateral occlusion were included in multivariate analysis (Table 3). For the MACCE endpoint, symptomatic status, diabetes and myocardial infarction, contralateral occlusion, symptomatic status and resection mostly in case of an elongated artery were each independently associated with a higher risk.

Coronary artery disease was not an independent risk factor, when it was used instead of myocardial infarction in multivariate analysis for the MACCE endpoint (HR 1.633, CI 0.901–2.960; *p* = 0.106). Of the 50 patients who suffered from MACCE, 12% (6) had none of our independent risk factors, 24% (12) had 1 factor, 40% (20) had a combination of 2 criteria and 24% (12) ≥ 3 factors. Analyzing the additive effect of independent factors similar to a score in a retrospective fashion by valuing the presence of each factor equivalently (one), the hazard ratio increases for presence of 2 factors from 1.90 (CI 1.07–3.33) to 2.73 (CI 1.33–5.62) for 3 factors, and to 17.1 (CI 5.3–55.2) for 4. ROC analysis supports the predictive power at an area under the curve of 0.66 (CI 0.578–0.751) with a balanced sensitivity and specificity at a cut of ≥ 2 factors.

### SAPPHIRE high risk and excluded patients

The relatively high event rate for both symptomatic and asymptomatic patients can be related to patients fulfilling SAPPHIRE high risk (SHR) and SAPPHIRE exclusion criteria (SE) (Table 4). Patients with none of the SAPPHIRE risk factors (SLR) had a MACCE rate of 4.7%. When comparing the SHR to the SLR patients, there was a significant difference (*n* = 178, HR 1.952, CI 1.032–3.696, *p* = 0.04, MACCE 9.0%) (Table 5). This effect persisted after adjustment for symptomatic status (HR for MACCE 2.069, CI 1.087–3.941, *p* = 0.027). Importantly, there were even fewer symptomatic patients in the SHR compared to the SLR groups (54.5% versus 68.3%, *p* = 0.001). Patients who would have been excluded from the SAPPHIRE study (SE) had a very high MACCE rate of 13.6% (HR 2.411, CI 1.235–4.078, *p* = 0.01 compared to all other patients). This effect was still seen after adjustment for symptomatic status (HR for MACCE 2.389, CI 1.22–4.66; *p* = 0.011).

**Table 4:** SAPPHIRE high risk (SHR) and exclusion (SE) criteria

Criteria for 178 SHR patients	*n* (%)	Criteria for 81 SE patients	*n* (%)
Age ≥ 80	97 (13.0)	Ischemic stroke in 48 h before surgery	2 (0.3)
Severe cardiac disease (NYHA III or IV)	64 (8.6)	Total occlusion of the carotid artery	4 (0.5)
Myocardial infarction 4 weeks preoperatively	5 (0.7)	Elective surgery within 30 days after CEA	19 (2.5)
Severe pulmonary dysfunction	4 (0.5)	Presence of an intraluminal thrombus	52 (7.0)
Contralateral carotid artery occlusion	51 (6.8)	Life expectancy less than one year	5 (0.7)
Previous radical neck surgery or radiation	4 (0.5)	Intracranial mass	3 (0.4)
Restenosis	3 (0.4)	–	

*CEA* carotid endarterectomy, *SAPPHIRE* the stenting and angioplasty with protection in patients at high risk for endarterectomy trial; (other criteria like intracranial aneurysm > 9 mm in diameter, need for more than two stents, history of bleeding disorder, percutaneous or surgical intervention planned within next 30 days, contralateral palsy of laryngeal nerve did not apply)

**Table 5:** Risk stratification with SAPPHIRE criteria adjusted for symptomatic status

Event	SLR (*n* = 489)		SHR (*n* = 178)		SE (*n* = 81)	
	*n*	%	*n*	%	*n*	%
MACCE	23	4.7	16	9.0	11	13.6
COX analysis for MACCE			SHR versus SLR		SE versus non SE	
HR			2.069		2.389	
CI			1.087–3.941		1.223–4.666	
*p*			0.027		0.011	

*CI* confidence interval, *HR* hazard ratio, *MACCE* Major Cardiac and Cerebrovascular Events, *p* level of significance, *SE* SAPPHIRE exclusion criteria, *SHR* SAPPHIRE high risk, *SLR* SAPPHIRE low risk (none of the SAPPHIRE risk factors)

By excluding the SAPPHIRE exclusion group, the overall MACCE rate was 5.8%, and the overall death and stroke rate was 4.9%. The death and stroke rate for symptomatic patients was 6.0% and it was 2.9% for asymptomatic patients.

Singular SHR and SE criteria were submitted to univariate regression analysis. The SHR criteria contralateral ICA occlusion and NYHA 3 or 4 showed a significant influence on MACCE and MI within 4 weeks before surgery a strong trend. Regarding the SE criteria, only intraluminal thrombus showed a significant influence (see Supplementary Table 2).

## Discussion

This prospective study of CEA substantiates that the high risk and particularly the exclusionary criteria used in the SAPPHIRE trial are strongly associated with MACCE from CEA similar to our recent findings that these exclusion criteria predict adverse outcomes following carotid artery stenting in clinical practice [21]. Patients who did not carry any SAPPHIRE high risk or exclusion criteria had better outcomes with a 30% reduced MACCE rate and a stroke and death rate matching guideline recommendations [5, 6]. The SAPPHIRE criteria have not been systematically evaluated for the MACCE rate at 30 days after CEA using contemporary standards. Retrospectively retrieved MACCE data limited to the intrahospital period after CEA lacking systematic testing for MI, did not show a higher MACCE rate in SAPPHIRE high-risk patients, however SAPPHIRE exclusion criteria were not analysed [23]. MACCE was only more frequent in the symptomatic SHR-group (9.3% versus 1.6%, *p* < 0.05) and postoperative MI showed a higher prevalence (3.1% versus 0.9%, *p* < 0.05) [23].

In the study presented, thirty five percent of patients fulfilled the SAPPHIRE study high risk (SHR) and exclusion (SE) criteria and these patients clearly demonstrated a higher event rate (SHR 9.0% and SE 13.6% MACCE versus 4.7% MACCE in average risk patients). Two of our independently associated risk factors (MI and contralateral occlusion) were also part of the SHR criteria, supporting their relevance besides the SE criteria. Therefore, an in-depth neurologic and cardiologic evaluation, neurovascular ultrasonography and cerebrovascular MR-imaging to assess SHR and SE criteria may be meaningful for neurovascular teams and interdisciplinary neurovascular boards deserving increased attention within stratification algorithms [5, 6]. Sixty-four percent of the patients, who suffered from MACCE, had a combination of ≥ 2 independent risk factors and a minority had none, disclosing a cumulation of risk factors and surmising an additive risk effect in the affected individuals. Within our cohort, the independent risk factors were found to have an additive effect on worse outcome upon retrospective analysis at a potentially relevant cut of ≥ 2 risk factors substantiating the clinical relevance at least in part. A possibly resulting score seeks to be evaluated in other populations. The important question, if symptomatic patients at an additionally severely increased risk fulfilling ≥ 2 risk factors or SE criteria should be operatively revascularized at all needed to be answered with a randomized controlled trial, which can hardly be ethically justified at least in severe stenosis of > 70%, that are prone to develop strokes in about 30% within the first weeks after the index event [22]. However, the comparison may be of interest for subgroups like patients with symptomatic moderate stenoses (50–69%), that showed an absolute stroke and death reduction upon CEA in the vulnerable phase within 2 weeks after the neurologic index event of 14% similar to the event rate of subgroups at high risk [22]. Presumably, outcome events have markedly decreased since then, as nowadays best medical treatment goals are more rigorous and drugs more effective [4, 5, 25]. Therefore, it remains speculative, if modern best medical treatment regimes like aggressive lipid lowering and dual antiplatelet aggregation treatment with aspirin and low dose novel oral anticoagulants may overtake the effectiveness of an invasive approach at least in subgroups with symptomatic stenosis, warranting new controlled trials [25]. As MI, SHR- and SE criteria predicted MACCE independent of the symptomatic status, it would be of interest to find out, if asymptomatic SE positive patients have a higher MACCE rate with or without an operation especially on longer term follow-up. However, the indications to operate on asymptomatic stenosis have decreased to low volumes in this country, leaving progression > 20%/6 months, emboligenic morphologies for CEA among other relative indications also necessitating comparative trials employing modern treatment regimes [4, 5].

Overall, the relatively high MACCE rate in this cohort was similar to the 5.6% periinterventional MACCE rate found in our previous evaluation of carotid artery stenting within a smaller cohort at the two institutions. However, the proportions of patients fulfilling SE criteria differed (20.5% CAS versus 10.3% of the CEA cohort) [21]. A relative doubling of the MACCE rate following CEA in the SE criteria positive subgroup compared to the average risk (SLR) and the SHR groups together corresponded to the twofold increase of the MACCE + TIA rate after stenting (SE 23% versus SLR + SHR 11.4%, HR 2.6; 95% CI 1.3–5.0; *p* = 0.007). In view of the heterogeneity of our CEA and CAS cohorts and due to the smaller size and low absolute stroke events of the stenting cohort a comparative head to head analysis is suggested between propensity matched larger populations.

Besides the high proportion of patients with SHR and SE criteria, our population contained patients with severe renal- and heart failure, both showing a strong trend for MACCE in our univariate analysis, patients with progressive strokes and with additional carotid or intracranial stenosis besides asymptomatic individuals with silent infarctions, all assumed to be risk factors for worse outcomes, reasoning exclusion from the majority of other prospective trials. This substantiates our very high cardiovascular risk structure in addition to the SAPPHIRE positive patients and may at least partially explain the overall increased MACCE in contrast to RCTs [1–3, 18, 19, 29].

Furthermore, we in particular highlight the importance of risk factors related to cardiac morbidity, as both prior myocardial infarction and diabetes were independently associated with 30-day MACCE. Strikingly, there are just a few studies assessing MACCE following CEA and, aside from an analysis using quality-based reporting, this is the first prospective registry study identifying the contemporary definition of prior MI as an independent predictor for 30-day MACCE [26].

The importance of MACCE was emphasized as perioperative MI was demonstrated to be a determinant of death during long-term follow up after major vascular surgery [20]. Similarly, the CREST trial showed a higher rate of postoperative MI after CEA than after CAS and a higher long-term mortality within this patient group [27]. The incidence of MI observed in our study (1.9%) was lower than in the CREST trial (2.4%) [18]. Several even most recent studies did not examine preoperative MI as a risk variable and analysed a combination of risk factors containing MI or used non-standard definitions of MI even within the endpoint, different from our CREST conform definitions [29–33]. Similar to our results they showed contralateral occlusion, diabetes and symptomatic status to be relevant predictors for their differently defined endpoints. In addition, age, smoking, intraluminal thrombus, siphon stenosis, elevated creatinine, history of atherosclerotic disease and severe haemorrhage have also been reported to predict “MACCE”-like outcomes independently [29–32]. Taken as a whole, a further important conclusion from our analysis is that significant coronary artery disease and the manifestation of myocardial infarction needs to be rigorously ruled out before CEA especially in patients with previously diagnosed CHD, NYHA ≥ 2 and diabetes found to be specifically associated with both, perioperative MI and MACCE. Therefore, the full spectrum of cardiodiagnostic modalities like laboratory markers for ischemia and heart failure, echocardiography including stress testing and coronary angiography needs to be adequately indicated preoperatively. Vice versa, the awareness for postoperative MI needs to be increased especially in patients not having received coronary angiography for example in neurologically symptomatic patients urgently needing CEA showing an increased pretest likelihood for CHD like the risk factors evaluated above. Furthermore, attention needs to be drawn towards the majority of NSTEMIs and the consequent need for frequent hsTroponin testing at least until day 3, when 75% of our patients were diagnosed with MI.

Furthermore, we assessed whether intraoperative and anatomic factors were associated with MACCE outcomes. Prior studies have reported that correcting elongated and kinked vessels is associated with worse outcome [34]. In our cohort resections were independently associated with an increased risk of MACCE and a subanalysis suggests that severely calcified and complex, progressively altered plaque morphologies were associated with more complicated reconstructions while elongation, kinking or coiling alone did not. Corresponding findings on duplex ultrasonography, computed tomography- or magnetic resonance angiography may, therefore, need to be comparatively analysed to find quantifiable imaging predictors, which we have begun prospectively in a systematic fashion.

### Limitations

As this study is a real-life cohort without a prespecified treatment protocol, there might have been slight differences in patient selection and CEA procedure and follow-up, although treatment was in conformity to active guidelines of the American Heart and Stroke Association and the Society of Vascular Surgeons. Given that the study only assessed patients treated at two centers, these practice patterns and outcomes may not be generally applicable to all settings. 5.6% of the patients did not complete the follow-up of 30 days and might have led to a selection bias. Nevertheless, taken together, the incremental value of this study is based on a prospective accumulation of data without the narrow selection bias found in RCTs.

## Conclusion

Carotid endarterectomy in a real-life large volume setting comprises a population at risk warranting specific attention regarding the prevention of MACCE that could be identified by the SAPPHIRE high risk and exclusion criteria, previous MI and diabetes besides established factors necessitating more extensive preoperative cerebral and cardiac diagnostics possibly including invasive coronary imaging besides postoperative surveillance for MI.

## Electronic supplementary material

Below is the link to the electronic supplementary material.

Supplementary file1 (DOCX 18 kb)

Supplementary file2 (DOCX 16 kb)

## References

[1] MRC Asymptomatic Carotid Surgery Trial (ACST) Collaborative Group (2004). Prevention of disabling and fatal strokes by successful carotid endarterectomy in patients without recent neurological symptoms: randomised controlled trial. Lancet.

[2] Barnett HJ, Taylor DW, Eliasziw M, Fox AJ, Ferguson GG, Haynes RB, Rankin RN, Clagett GP, Hachinski VC, Sackett DL, Thorpe KE, Meldrum HE, Spence JD (1998). Benefit of carotid endarterectomy in patients with symptomatic moderate or severe stenosis. N Engl J Med.

[3] ECST collaborators (1998). Randomised trial of endarterectomy for recently symptomatic carotid stenosis: final results of the MRC European Carotid Surgery Trial (ECST). Lancet.

[4] Abbott AL (2009). Medical (nonsurgical) intervention alone is now best for prevention of stroke associated with asymptomatic severe carotid stenosis: results of a systematic review and analysis. Stroke.

[5] Aboyans V, Ricco JB, Bartelink MEL, Bjorck M, Brodmann M, Cohnert T, Collet JP, Czerny M, De Carlo M, Debusa S, Espinola-Klein C, Kahan T, Kownator S, Mazzolai L, Naylora AR, Roffi M, Rotherb J, Sprynger M, Tendera M, Tepe G, Venermoa M, Vlachopoulos C, Desormais I (2018). 2017 ESC guidelines on the diagnosis and treatment of peripheral arterial diseases. Eur Heart J.

[6] Brott TG, Halperin JL, Abbara S, Bacharach JM, Barr JD, Bush RL, Cates CU, Creager MA, Fowler SB, Friday G, Hertzberg VS, McIff EB, Moore WS, Panagos PD, Riles TS, Rosenwasser RH, Taylor AJ, Jacobs AK, Smith SC, Anderson JL, Adams CD, Albert N, Buller CE, Creager MA, Ettinger SM, Guyton RA, Halperin JL, Hochman JS, Hunt SA, Krumholz HM, Kushner FG, Lytle BW, Nishimura RA, Ohman EM, Page RL, Riegel B, Stevenson WG, Tarkington LG, Yancy CW (2011). 2011 ASA/ ACCF/ AHA/ AANN/ AANS/ ACR/ ASNR/ CNS/ SAIP/ SCAI/ SIR/ SNIS/ SVM/ SVS guideline on the management of patients with extracranial carotid and vertebral artery disease. J Am Coll Cardiol.

[7] Kammler J, Blessberger H, Lambert T, Kellermair J, Grund M, Nahler A, Lichtenauer M, Schwarz S, Reiter C, Steinwender C, Kypta A (2017). In-stent restenosis after interventional treatment of carotid artery stenoses: a long-term follow-up of a single center cohort. Clin Res Cardiol.

[8] Haeusler KG, Gröschel K, Köhrmann M, Anker SD, Brachmann J, Böhm M, Diener HC, Doehner W, Endres M, Gerloff C, Huttner HB, Kaps M, Kirchhof P, Nabavi DG, Nolte CH, Pfeilschifter W, Pieske B, Poli S, Schäbitz WR, Thomalla G, Veltkamp R, Steiner T, Laufs U, Röther J, Wachter R, Schnabel R (2018). Expert opinion paper on atrial fibrillation detection after ischemic stroke. Clin Res Cardiol.

[9] Rothwell PM, Slattery J, Warlow CP (1997). Clinical and angiographic predictors of stroke and death from carotid endarterectomy: systematic review. BMJ.

[10] Hannan EL, Popp AJ, Feustel P, Halm E, Bernardini G, Waldman J, Shah D, Chassin MR (2001). Association of surgical specialty and processes of care with patient outcomes for carotid endarterectomy. Stroke.

[11] Kragsterman B, Logason K, Ahari A, Troëng T, Parsson H, Bergqvist D (2004). Risk factors for complications after carotid endarterectomy—a population-based study. Eur J Vasc Endovasc Surg.

[12] Goodney PP, Likosky DS, Cronenwett JL (2008). Factors associated with stroke or death after carotid endarterectomy in Northern New England. J Vasc Surg.

[13] Halm EA, Tuhrim S, Wang JJ, Rockman C, Riles TS, Chassin MR (2009). Risk factors for perioperative death and stroke after carotid endarterectomy: results of the New York carotid artery surgery study. Stroke.

[14] Halm EA, Hannan EL, Rojas M, Tuhrim S, Riles TS, Rockman CB, Chassin MR (2005). Clinical and operative predictors of outcomes of carotid endarterectomy. J Vasc Surg.

[15] Gupta PK, Pipinos II, Miller WJ, Gupta H, Shetty S, Johanning JM, Longo GM, Lynch TG (2011). A population-based study of risk factors for stroke after carotid endarterectomy using the ACS NSQIP database. J Surg Res.

[16] Bennett KM, Scarborough JE, Shortell CK (2015). Predictors of 30-day postoperative stroke or death after carotid endarterectomy using the 2012 carotid endarterectomy-targeted American College of Surgeons National Surgical Quality Improvement Program database. J Vasc Surg.

[17] Yadav JS, Wholey MH, Kuntz RE, Fayad P, Katzen BT, Mishkel GJ, Bajwa TK, Whitlow P, Strickman NE, Jaff MR, Popma JJ, Snead DB, Cutlip DE, Firth BG, Ouriel K (2004). Protected carotid-artery stenting versus endarterectomy in high-risk patients SAPPHIRE. N Engl J Med.

[18] Brott TG, Hobson RW, Howard G, Roubin GS, Clark WM, Brooks W, Mackey A, Hill MD, Leimgruber PP, Sheffet AJ, Howard VJ, Moore WS, Voeks JH, Hopkins LN, Cutlip DE, Cohen DJ, Popma JJ, Ferguson RD, Cohen SN, Blackshear JL, Silver FL, Mohr JP, Lal BK, Meschia JF (2010). Stenting versus endarterectomy for treatment of carotid-artery stenosis. N Engl J Med.

[19] ICSS collaborators (2010). Carotid artery stenting compared with endarterectomy in patients with symptomatic carotid stenosis (International Carotid Stenting Study (ICSS)). Lancet.

[20] Landesberg G, Shatz V, Akopnik I, Wolf YG, Mayer M, Berlatzky Y, Weissman C, Mosseri M (2003). Association of cardiac troponin, CK-MB, and postoperative myocardial ischemia with long-term survival after major vascular surgery. J Am Coll Cardiol.

[21] Macharzina RR, Claus C, Messé SR, Boehme T, Vach W, Winker T, Rastan A, Beschorner U, Noory E, Neumann FJ, Zeller T (2015). History of transient ischaemic attack, myocardial infarction and hyperlipidaemia affects outcome following carotid artery stenting. Eurointervention.

[22] Sacco RL, Adams R, Albers G, Alberts MJ, Benavente O, Furie K, Goldstein LB, Gorelick P, Halperin J, Harbaugh R, Johnston SC, Katzan I, Kelly-Hayes M, Kenton EJ, Marks M, Schwamm LH, Tomsick T (2006). Guidelines for prevention of stroke in patients with ischemic stroke or transient ischemic attack. Stroke.

[23] Mozes G, Sullivan TM, Torres-Russotto DR, Bower TC, Hoskin TL, Sampaio SM, Gloviczki P, Panneton JM, Noel AA, Cherry KJ (2004). Carotid endarterectomy in SAPPHIRE-eligible high-risk patients: implications for selecting patients for carotid angioplasty and stenting. J Vasc Surg.

[24] Rothwell PM, Eliasziw M, Gutnikov SA, Warlow CP, Barnett HJ (2004). Endarterectomy for symptomatic carotid stenosis in relation to clinical subgroups and timing of surgery. Lancet.

[25] Sharma M, Hart RG, Connolly SJ, Bosch J, Shestakovska O, Ng KKH, Catanese L, Keltai K, Aboyans V, Alings M, Ha JW, Varigos J, Tonkin A, O'Donnell M, Bhatt DL, Fox K, Maggioni A, Berkowitz SD, Bruns NC, Yusuf S, Eikelboom JW (2019). Stroke outcomes in the COMPASS trial. Circulation.

[26] Biller J, Feinberg WM, Castaldo JE (1998). Guidelines for carotid endarterectomy. A statement for healthcare professionals from a special writing group of the stroke council. American Heart Association Circulation.

[27] Blackshear JL, Cutlip DE, Roubin GS, Hill MD, Leimgruber PP, Begg RJ, Cohen DJ, Eidt JF, Narins CR, Prineas RJ, Glasser SP, Voeks JH, Brott TG (2011). Myocardial infarction after carotid stenting and endarterectomy: results from the carotid revascularization endarterectomy versus stenting trial. Circulation.

[28] Nicolaides AN, Shifrin EG, Bradbury A, Dhanjil S, Griffin M, Belcaro G, Williams M (1996). Angiographic and duplex grading of internal carotid stenosis: can we overcome the confusion?. J Endovasc Surg.

[29] Stoner MC, Abbott WM, Wong DR, Hua HT, Lamuraglia GM, Kwolek CJ, Watkins MT, Agnihotri AK, Henderson WG, Khuri S, Cambria RP (2006). Defining the high-risk patient for carotid endarterectomy: an analysis of the prospective National Surgical Quality Improvement Program database. J Vasc Surg.

[30] Goldstein LB, McCrory DC, Landsman PB, Samsa GP, Ancukiewicz M, Oddone EZ, Matchar DB (1994). Multicenter review of preoperative risk factors for carotid endarterectomy in patients with ipsilateral symptoms. Stroke.

[31] Goldstein LB, Samsa GP, Matchar DB, Oddone EZ (1998). Multicenter review of preoperative risk factors for endarterectomy for asymptomatic carotid artery stenosis. Stroke.

[32] van Lammeren GW, Catanzariti LM, Peelen LM, de Vries JP, de Kleijn DP, Moll FL, Pasterkamp G, Bots ML (2012). Clinical prediction rule to estimate the absolute 3-year risk of major cardiovascular events after carotid endarterectomy. Stroke.

[33] Volkers EJ, Algra A, Kappelle LJ, Becquemin JP, de Borst GJ, Brown MM, Bulbulia R, Calvet D, Eckstein HH, Fraedrich G, Gregson J, Halliday A, Hendrikse J, Howard G, Jansen O, Roubin GS, Bonati LH, Brott TG, Mas JL, Ringleb PA, Greving JP (2019). Safety of carotid revascularization in patients with a history of coronary heart disease. Stroke.

[34] Illuminati G, Ricco JB, Caliò FG, D’Urso A, Ceccanei G, Vietri F (2008). Results in a consecutive series of 83 surgical corrections of symptomatic stenotic kinking of the internal carotid artery. Surgery.

