# Supplementary material for: The SAPPHIRE criteria, history of myocardial infarction and diabetes predict adverse outcomes following carotid endarterectomy similar to stenting
Source: Clin Res Cardiol. 2019 Sep 25;109(5):589–98. doi: 10.1007/s00392-019-01546-3 (PMC7182626; doi:10.1007/s00392-019-01546-3)
Supplement: Supplementary file 1 — Supplementary file1 (DOCX 18 kb) [file 392_2019_1546_MOESM1_ESM.docx]

| Supplementary Table 1. Univariate Cox analysis to identify risk factors of MACCE at 30 days (n=748) | | | | |
| --- | --- | --- | --- | --- |
|  | **Event-rate of patients negative / positive for criteria, n (%)** | **HR** | **95%-CI** | ***p*** |
| Age [y, continuous] | - | 1.019 | 0.988-1.052 | 0.235 |
| Age ≥80y | 42 (6.4) / 8 (8.2) | 1.291 | 0.606-2.750 | 0.508 |
| Female | 37 (6.6) / 13 (7.1) | 1.078 | 0.573-2.028 | 0.815 |
| Symptomatic < 6 months contralateral | 13 (5.0) / 37 (7.6) | 1.547 | 0.823-2.911 | 0.18 |
| Body-Mass-Index | - | 0.972 | 0.907-1.042 | 0.422 |
| Diabetes | 27 (5.1) / 23 (10.6) | 2.127 | 1.219-3.709 | 0.008 |
| Hypertension | 3 (3.3) / 47 (7.2) | 2.213 | 0.689-7.109 | 0.182 |
| HLP on statin treatment | 9 (4.8) / 41 (7.3) | 1.536 | 0.746-3.160 | 0.244 |
| Current or past smoking | 32 (8.0) / 18 (5.2) | 1.554 | 0.872-2.768 | 0.135 |
| Coronary heart disease | 23 (5.1) / 27 (9.1) | 1.793 | 1.028-3.127 | 0.040 |
| Heart failure (NYHA III, IV) | 42 (6.1) / 8 (12.5) | 2.067 | 0.971-4.404 | 0.060 |
| Renal failure (NKF III, IV) | 30 (5.8) / 20 (9.3) | 1.638 | 0.930–2.884 | 0.087 |
| Atrial fibrillation | 47 (7.0) / 2 (3.6) | 0.518 | 0.126-2.134 | 0.363 |
| Myocardial infarction | 34 (5.5) / 16 (12.3) | 2.295 | 1.267-4.157 | 0.006 |
| Family history of cardiovascular disease | 40 (7.1) / 10 (5.5) | 0.768 | 0.384-1.537 | 0.456 |
| Antiplatelet therapy | 7 (10.6) / 43 (6.3) | 0.589 | 0.265-1.310 | 0.194 |
| High grade stenosis | 6 (4.7) / 44 (7.1) | 1.522 | 0.648-3.570 | 0.335 |
| Contralateral ICA stenosis | 40 (6.3) / 10 (9.4) | 1.532 | 0.766-3.063 | 0.228 |
| Contralateral ICA occlusion | 44 (6.3) / 6 (11.8) | 1.927 | 0.821-4.522 | 0.132 |
| **Procedure related factors** | |  |  |  |
| Eversion endarterectomy | 19 (6.6) / 30(6.6) | 1.011 | 0.569-1.795 | 0.971 |
| Resection | 40 (5.9) / 9 (13.2) | 2.300 | 1.116-4.074 | 0.024 |
| Intraoperative carotid bypass | 42 (7.7) / 7 (3.7) | 0.472 | 0.212-1.051 | 0.066 |
| Operation time (≥75^th^ percentile, 140 min) | 37 (6.5) / 13 (7.3) | 1.141 | 0.606-2.146 | 0.683 |
| CEA, carotid endarterectomy; CI, confidence interval; HLP, hyperlipoproteinaemia; HR, hazard ratio; ICA, internal carotid artery; MACCE, Major Adverse Cardiac and Cerebrovascular Events; NKF, National Kidney Foundation; NYHA, New York Heart Association heart failure scale; *p,* value of significance. | | | | |
