# Supplementary material for: The SAPPHIRE criteria, history of myocardial infarction and diabetes predict adverse outcomes following carotid endarterectomy similar to stenting
Source: Clin Res Cardiol. 2019 Sep 25;109(5):589–98. doi: 10.1007/s00392-019-01546-3 (PMC7182626; doi:10.1007/s00392-019-01546-3)
Supplement: Supplementary file 2 — Supplementary file2 (DOCX 16 kb) [file 392_2019_1546_MOESM2_ESM.docx]

| Supplementary Table 2. Univariate Cox analysis to identify the risk of singular SAPPHIRE criteria for MACCE at 30 days (n=748) | | | | |
| --- | --- | --- | --- | --- |
|  | **Event-rate of patients negative / positive for criteria, n (%)** | HR | 95%-CI | *p* |
| **SHR criteria (excluding the SE patients)** | **n = 39 events** |  |  |  |
| Age ≥80 | 32 (5.5%)/  7 (7.8%) | 1.405 | 0.620-3.184 | 0.415 |
| Contralateral ICA occlusion | 33 (5.3%)/  6 (12.8%) | 2.511 | 1.052-5.993 | 0.038 |
| Heart failure  (NYHA 3 or 4) | 33 (5.3%)/  6 (12.5%) | 2.397 | 1.004-5.720 | 0.04 |
| MI within 4 weeks before operation | 38 (5.7%)/  1 (33.3%) | 7.129 | 0.978-51.961 | 0.05 |
| Severe pulmonary dysfunction | 38 (5.7%)/  1 (25.0%) | 5.004 | 0.687-36.462 | 0.112 |
| Radical neck surgery or radiation | 39 (5.9%)/  0 (0%) | n.a. | n.a. | n.a. |
| Restenosis | 39 (5.9%)/  0 (0%) | n.a. | n.a. | n.a. |
| **SE criteria** |  | | | |
| Ipsilateral occlusion | 49 (6.6%)/  1 (25.0%) | 4.365 | 0.602-31.61 | 0.145 |
| Stroke <48h before | 50 (6.7%)/  0 (0%) | n.a. | n.a. | n.a. |
| Intracranial mass | 50 (6.7%)/  0 (0%) | n.a. | n.a. | n.a. |
| Elective surgery <30 days before | 48 (6.6%)/  2 (10.5%) | 1.655 | 0.402-6.809 | 0.485 |
| Life expectancy <1 year | 50 (6.7%)/  0 (0%) | n.a. | n.a. | n.a. |
| Intraluminal thrombus | 37 (5.4%)/  9 (17.3%) | 3.351 | 1.617-6.943 | 0.001 |
| CI, confidence interval; HR, hazard ratio; ICA, internal carotid artery; MACCE, Major Adverse Cardiac and Cerebrovascular Events; MI, myocardial infarction; n.a., not applicable; *p,* value of significance; SE, SAPPHIRE exclusion; SHR, SAPPHIRE high risk. | | | | |
